# Exosomes: A Source for New and Old Biomarkers in Cancer

**DOI:** 10.3390/cancers12092566

**Published:** 2020-09-09

**Authors:** Mariantonia Logozzi, Davide Mizzoni, Rossella Di Raimo, Stefano Fais

**Affiliations:** Department of Oncology and Molecular Medicine, Istituto Superiore di Sanità, Viale Regina Elena 299, 00161 Rome, Italy; mariantonia.logozzi@iss.it (M.L.); davide.mizzoni@iss.it (D.M.); rossella.diraimo@iss.it (R.D.R.)

**Keywords:** old tumor biomarkers, circulating exosomes, exosome biomarkers, biological fluids, non-invasive tools

## Abstract

**Simple Summary:**

The follow-up of patients with tumors needs new or at least more reliable biomarkers to avoid invasive approaches. Unfortunately, the existing biomarkers too often have generated more problems than having proven to be sufficiently helpful for the clinical oncologists. Very recently, translational research has focused on extracellular vesicles, with size ranging between micro to nano as potential sources of new tumor biomarkers. In particular, nanovesicles (called exosomes) that are variably released from virtually all cells, have shown to be a potential source of new tumor biomarkers but also a preferential delivery system for well-known biomarkers, such as PSA and CEA. The clinical data supporting this new research area are, unfortunately, very few, but the existing reports are very encouraging. We review and discuss the existing literature supporting a key role of exosomes as a source of tumor biomarkers, together with some unexpected discoveries.

**Abstract:**

Clinical oncology needs reliable tumor biomarkers to allow a follow-up of tumor patients who do not necessarily need invasive approaches. To date, the existing biomarkers are not sufficiently reliable, and many of them have generated more problems than facilitating the commitment of clinical oncologists. Over the last decades, a broad family of extracellular vesicles, with size ranging between micro to nano, has been raised as a new hope for potential sources of new tumor biomarkers. However, while knowledge in the field is increasing, we do not currently have definitive information allowing a clinical use of extracellular vesicles in cancer clinics. Recent evidence provides new perspective in clinical oncology, based on data showing that circulating nanovesicles called exosomes may represent a valuable source of tumor biomarkers. In this review, we discuss the existing clinical data supporting a key role of exosomes as a source of tumor biomarkers, including proteins and miRNAs, but also discuss the importance of the expression of known tumor biomarkers when expressed on exosomes.

## 1. What Exosomes Are

Exosomes are 40–180 nm extracellular vesicles that are released from virtually all cells under normal and pathological conditions [1,2,3,4,5]. They form by membrane invagination of late endosomes and are released in the extracellular microenvironment after multivesicular bodies (MVBs) fusion with the plasma membrane [1,2,6,7]. Thus, the exosomes proteins’ make up derives from a cascade of internal vesicle fusion, MVB formation, and their fusion with the plasma membrane makes. Exosomes express specific markers of the endosomal pathway, such as tetraspanins (CD63, CD9, and CD81), but also heat shock proteins (HSP70) and proteins from the Rab family, Tsg101 and Alix, which are not detectable in other types of vesicles of similar size [1,2,8]. For similar reasons, exosomes may express markers acquired during the process of internal vesicles’ fusion with the plasma membrane, thus witnessing the cellular source [1,2]. We know that hematopoietic cells (B cells, T cells, dendritic cells, mast cells, and platelets), intestinal epithelial cells, Schwann cells, neuronal cells, adipocytes, fibroblasts (NIH3T3), and tumor cells release vesicles extracellularly [8,9]. However, exosomes have a characteristic lipid bilayer composition of their membrane, and they contain a cargo of nucleic acids, including DNAs, messenger RNAs (mRNAs), and microRNAs (miRNAs) [1,2]. Recently, it has been shown that exosomes deliver proteins exerting a functional activity. From a functional point of view, extracellular vesicles (EVs) were originally suggested to be involved in the removal of unnecessary molecules that are poorly degraded by the intracellular lysosomal system [10]. Indeed, exosomes are not only cell ‘cleaners’, but crucial actors in cell-to-cell communication [1,2,3,5,11,12,13,14,15]. They are detectable in virtually all biological fluids, including plasma, urine, cerebrospinal fluid, epididymal fluid, amniotic fluid, malignant and pleural effusions of ascites, bronchoalveolar lavage fluid, synovial fluid, and breast milk, suggesting a that EVs may have a critical role in connecting tissues, organs and compartments of our body [1,9,16,17,18], but also transferring infectious agents including viruses and pathological prion proteins [13,19].

Knowledge of EVs is rapidly increasing and what we are learning is extremely challenging. In fact, the available data support a key role of this broad family of vesicles in both the homeostasis of our body and the pathophysiology of the vast majority of human diseases, including cancer, thus representing a valuable source of disease biomarkers [1,2,20,21,22,23,24,25,26,27,28,29,30].

Presently, the protein cargo of EVs and exosomes that have been purified from human body fluids have shown some level of variability, due to both the broad spectrum of cellular sources and the inter-individual variability [20,31,32,33]. The choice of the exosome source is pivotal to obtain reliable and reproducible results, considering the different biophysical and chemical properties of each biological fluid. It is, in fact, mandatory that, despite the possible lipoproteins contamination, plasma and serum are by far the body fluids witnessing a systemic situation; while, for instance, urine and stool may represent a source of exosomes from the urinary tract and the gut, respectively [1,2,34]. Moreover, the plasma is considered the most suitable source of exosomes being deprived of fibrinogen, thus allowing the best exosome recovery in terms of number and cargo (proteins and RNA) [1,9,22,34].

Thanks to a general improvement of the technical equipment to analyze EVs, as represented by nanoparticle tracking analysis (NTA), immune captured based technologies, and nanoscale flow cytometry (NFC), we can now obtain reliable quantitative and qualitative information potentially useful for future clinical application [16,20,35,36].

## 2. Isolation Methods of Plasma-Derived Exosomes

A consensus agreement on a standard method for isolation of plasma-derived exosomes still lacks actually, while many approaches have been proposed in the case of cell culture supernatants. However, ultracentrifugation is currently the widely used technique for exosomes isolation; it is based on the differential sedimentation rate of particles according to their size, shape, and density. Although this technique is time-consuming and raising some problems on the vesicle integrity, there is a general agreement supporting its use is the most versatile between the available methods, and probably the one providing the most complete extracellular vesicle yield independently from the source [2,33,37,38]. Another method that is used is an iodixanol/sucrose gradient or sucrose density cushion protocol (commonly defined as the density gradient ultracentrifugation). However, while it allows better purification of the EVs population, this technique leads to a great loss of nanovesicles during fractionation; and in plasma samples, it does not allow for the satisfactory separations of vesicles from apolipoproteins and blood HDL [39]. Size exclusion chromatography (SEC) has become a popular technique to purify exosomes from body fluids, in particular from plasma. It is a customizable procedure using different types of matrices/resins commercially available to enhance pick resolution of particles (increase in column length) of the following spectroscopic analysis, or concentrate samples from a large volume (increase in column diameter) [38]. SEC isolation excludes albumin from the exosome separations, but on the other side, it, together of being cost and time expensive, has shown to be not reliable as a technique to be translated into the clinical use, in as much as it provided variable results on the purity of samples between different laboratories [39].

Moreover, SEC leads to the formation of aggregates that affects the quality of the product, as it has been shown with all the methodologies based on polymeric precipitation mixtures [39]. Exosomes can be isolated from human body fluids by immunocapture or immunoaffinity, as well. These methods provide a methodology aimed at immunocapturing exosomes on antibodies attached to either plastic wells or immunomagnetic beads or chromatographic matrices or microfluidic platforms, all based on the specific recognition of exosomal membrane marker by monoclonal antibodies [33,37]. There is a general consensus on the reliability of this approach with a high potentiality for clinical application [37,38]. It currently has some limitations, mostly due to the low exosome yield and some problems in eluting the complexes exosomes-antibodies and some level of non-specific binding of other micro/nanocorpes to the substrates [33,37,38]. A further method that appears promising is the exosome purification, based on microfluidics allowing a better purity of exosomes isolation, in a very short time [33]. However, it did not show an ideal performance in terms of the number of EVs/exosomes obtained, and presently lacks an acceptable level of standardization [33]. In summary, while it is not perfect, the best approach is to use ultracentrifugation with implementation with immunocapture-based techniques. Of course, the goal of future techniques should be to set up methodologies that need to be validated—and are, hopefully, adaptable to all the clinical laboratories worldwide.

## 3. A Role of Exosomes in Cancer Progression and Metastasis

Malignant cancers have some phenotypes in common, including hypoxia, low nutrient supply, and extracellular acidosis [40,41,42,43]. Recently, EVs (especially exosomes) have emerged as an alternative mediator of cell-to-cell communication within the tumor microenvironment (TME) and cancer metastasis [41,42,43]. A marked increase in the exosomes, released under acidic pH (6.5) has been shown as compared to a physiological pH (7.4) [4]. This occurred independently from the tumor histotype, meaning that prostate cancer, melanoma, osteosarcoma, breast, and colorectal carcinoma-derived cell lines all showed an increased exosomes, released when cultured at acidic condition [4]. Moreover, the exosomes released at low pH showed always a smaller size as compared to those released at pH 7.4, which showed a more heterogeneous size [4]. The increase in the exosome release at in vitro acidic condition was comparable to the increased plasmatic exosome levels measurable in prostate cancer patients as compared to different controls, including inflammatory conditions, such as patients with benign prostate hypertrophy or healthy individuals [35]. However, there is increasing evidence that argues tumor microenvironmental acidity may actively participate in determining an increased release of exosomes in cancer conditions [5]. In fact, exosomes play a key role in tumor growth and metastasis through the formation of tumor niches in target organs together with inducing malignant transformation in resident mesenchymal stem cells [3,14,44,45].

The ensemble of these data shows that exosomes have a prominent role in favoring both the growth of primary tumors and their metastatic spread [44,45]. However, some data support a role of exosomes as primary actors in a recent Darwinian-like theory on tumor formation, hypothesizing that tumor progression passes through a microenvironment-determined progressive selection of cells with very high adaptive features—in turn, allowing tumors to live and grow in a very hostile microenvironment [46,47,48,49]. In fact, the experimental evidence supports a key role of the acidic tumor milieu in boosting exosome release from malignant cells [5]. It appears, therefore, conceivable that the exosome hyperproduction by tumor cells may be induced by the toxic microenvironment that probably selects cells that use an extracellular release of vesicles to eliminate toxic molecules to prevent their intracellular accumulation. Recent evidence suggests that exosomes released in acidic condition express ions transporters, such as Carbonic Anhydrase IX (CA IX), that on exosomes exerts a full enzymatic function [50]. A further investigation has shown that increased CA-related enzymatic function is measurable in plasmatic exosomes from cancer patients [51], suggesting that CA-IX expression and activity in plasmatic exosomes preparation may represent a new valuable cancer biomarker in the near future [20,52].

The versatile cargo of EVs, on the one hand, allows them to play a multitude of roles within the tumor microenvironment; on the other hand, it renders EVs a very promising source of tumor biomarkers [22,53], including protein, lipids, and a series of nucleic acids (i.e., DNAs, mRNAs, and miRNA) [17,21]. The harsh microenvironmental conditions of hypoxia, acidity, and low nutrient supply are responsible for the increased exosome release by tumor cells, as well as the hyperexpression of known tumor markers, such as PSA [35], and proteins related to ion/proton transport (e.g., V-ATPase, CA-IX) [50].

An issue that deserves some discussion is the increase in the number of circulating exosomes, that while in vitro related to the acidic pH, has been shown into the plasma of tumor patients as well [35]. This was hypothesized in 2017 [22] on the basis of clinical data obtained using an immunocapture-based technique [16] and confirmed with different techniques in tumor patients and independently from tumor histologies [4,35,54]. Moreover, pre-clinical in vivo experiments, reported a direct correlation between the tumor mass and the levels of plasmatic exosomes [16]. This was supported by some clinical reports showing that the surgical removal of the primary tumor has led to a dramatic drop down of the plasmatic exosome levels [55,56]. This suggests that the amount of circulating exosome levels may represent a way to monitor the effectiveness of both medical and surgical therapies, and also extremely useful in the patients’ follow-up. On the other hand, it appears conceivable that this huge amount of circulating tumor exosomes may represent a real danger for cancer patients, given the role of exosomes in tumor metastasis [3,14], also suggesting that therapies aimed at reducing exosome production by tumors may represent a goal of future anti-tumor treatments.

## 4. The Clinical Relevance of Exosomes as Biomarkers of Cancer

Research on exosomes as a source of tumor markers, covering several cancer types, is increasing (Table 1); nevertheless, only a few exosome-based diagnostic assays are currently available for clinical use.

An immunocapture-based ELISA (IC-ELISA) test pioneered an attempt to quantify and characterize plasmatic exosomes [16]. With this approach, it has been shown that CD63+ and CAV1+ plasma exosomes levels were significantly higher in melanoma patients as compared to healthy donors [16]. On the basis of the early described test, the IC-ELISA has been recently modified and compared with other emerging technologies, such as NTA and NFC [35]. The ensemble of these three techniques confirmed that under acidic condition, cancer cells released an increased number of exosomes [35] and that this was a common feature of virtually all cancers [4]. In the same study, both IC-ELISA and NFC showed that in acidic conditions, human prostate cancer cells released increased amounts of exosomes expressing PSA [35]. However, this approach was exploited in a prospective clinical study in prostate cancer patients, as compared to both patients with benign prostate hypertrophy (BPH) and healthy controls [36]. The results showed that the levels of plasmatic exosomes expressing PSA were significantly increased in patients with prostate cancer, with significantly higher sensitivity and specificity as compared to the standard serum PSA in the same patients [36]. A careful statistical analysis, comparing the serum PSA to the exosome PSA levels (as determined by either IC-ELISA or NFC), showed that the exosome-related measures are significantly correlated, thus reflecting the same biological phenomenon, but also that serum PSA and exosome PSA are independent values [36]. This confirmed the impossibility of serum PSA to discriminate Pca from BPH patients, while the plasmatic levels of exosomes expressing PSA clearly distinguished not only cancer patients from healthy individuals but cancer patients from patients with a non-tumor condition, such as BPH [36]. This is a result of paramount importance, inasmuch as serum PSA determination, while it is currently used worldwide for Pca early diagnosis and clinical follow-up, has sadly shown a high number of false positives and false negatives with many unwanted consequences [85].

These data highly support the use of IC-based technologies for both characterization and quantification of circulating exosomes, providing potential new sources of clinical biomarkers for screening tests, diagnosis, and follow-up of cancer patients, and possibly also patients with other diseases. IC-ELISA should deserve very much attention for several reasons including: (i) It is noninvasive; (ii) it is rapid, affordable, specific, quantitative, and versatile (easily extendable to other markers/conditions); it requires small amounts of sample and has multiple readouts; (iv) it allows both screenings and follow-up applications; and lastly (v) it can be translated with reasonable costs to all the research and clinical laboratories worldwide. Most of all, IC-ELISA provides the possibility of screening multiple markers within the same sample, and therefore, the possibility to explore in the same plasma sample the expression on exosomes of know tumor biomarkers, surrogate tumor biomarkers, and hopefully new tumor biomarkers.

## 5. Clinical Data on the Plasmatic Exosome Levels and the Hyperexpression of Known Tumor Biomarkers

As specified above, a current limitation of exosome diagnostic applications is the lack of standardization for methods concerning both exosome isolation and further analysis [33,37,38]. However, despite a number of commercially available assays for exosome isolation providing fast protocols, the approach allowing the best yield still requires ultracentrifugation protocols of large volumes [2,33,37,38]. Another important concern derives from the evidence is that the vast majority of exosome purification from human body fluids contain both the heavy and light chains of the immunoglobulins—this is intriguing information, but at the same time represents a great limit to perform reliable qualitative approaches, such as Western blot analysis [2,33,37].

By using an immunocapture-based assay, it was shown that patients with advanced melanoma patients (stage III and IV) had higher plasmatic exosomes levels as compared to healthy donors, with the best results with exosomes expressing caveolin-1 [16]. These data suggested the ability of exosomes-associated caveolin-1 to detect advanced melanoma more effectively than the Lactate dehydrogenase (LDH) serum levels commonly used in the follow-up of melanoma patients [16]. These findings are supported by recent studies showing that in melanoma patients, MIA and S100B-positive exosomes were significantly higher than in healthy controls [58] and that the amount of serum exosomes from patients with different hematological malignancies is significantly higher than in healthy individuals [84]. This last study identified markers on circulating nanovesicles reflecting their cellular source, including CD19 in B cell neoplasm, CD38 in multiple myeloma, CD13 in myeloid tumors, and CD30 in Hodgkin’s lymphoma [84].

A clinical study has shown that patients with Glioblastoma (GBM) have higher EVs plasma levels and that the EVs levels significantly decrease after surgical resection and raise again at recurrence [54], with very promising implication for therapy response, and monitoring.

According to these findings, a study has shown an increase of exosome levels in the urine of prostate cancer (PCA) patients using a time-resolved fluorescence immunoassay [86]. A clinical study performed in patients with colorectal cancer showed that the levels of plasmatic exosomes were statistically higher than in healthy controls [75]. A study, published in 2005, has shown that plasmatic exosomes from colon cancer patients expressed carcinoembryonic antigen (CEA), together with death receptors ligands [77]. In prostate cancer patients, the number of plasmatic exosomes, measured by NTA, was higher than in the plasma of healthy controls [60]. Recently, a milestone study has shown that glypican-1 (GPC1) positive exosomes were detectable in the serum of patients with pancreatic cancer with a high level of specificity and sensitivity, as compared to both healthy subjects and patients with a benign pancreatic disease [69]. However, the same study showed that high levels of GPC1 on exosomes can be detected in breast cancer patients as well [69], suggesting that exosome GPC1 is not a tumor-specific marker.

The ensemble of these clinical reports suggests the use of plasmatic exosomes quantification as a new and suitable method for the clinical follow-up of cancer patients. In further supporting this hypothesis, it has been shown that the effect of the treatment with Imatinib in patients with gastrointestinal stromal tumor (GIST) may be monitored by measuring the plasmatic levels of EVs before and after treatment [87]. Thus, suggesting that the effectiveness of a therapy may be evaluated by measuring the EVs levels in cancer patients. A recent clinical study performed in patients with Oral Squamous Cell Carcinoma has shown that surgical treatment induced a dramatic reduction of the plasmatic levels of exosomes expressing CD63 as early as one week after resection, as assessed by IC-ELISA [56]. A pre-clinical study has shown that treatment with proton pump inhibitors of a xenograft model of human melanoma induced a significant reduction of plasmatic EV levels consistent with a reduction of the tumor size [88]. This approach may be used in evaluating the effectiveness of radiotherapy as well [89].

Probably the most convincing pre-clinical evidence in this sense is that showing a direct correlation between exosome levels and the tumor size [16], suggesting that circulating exosomes in cancer patients may represent roughly an esteem of the tumor mass, including both the primary tumor and the metastasis.

A crucial issue is that the assessment of EVs levels in cancer patients may be highly improved if implemented with highly specific markers. To this purpose, it is mandatory to carry on with studies looking at new EVs-related tumor biomarkers, including proteins, lipids, and nucleic acids. Some clinical studies have shown that tumor patients’ exosomes express higher levels of some known tumor markers such as CEA in colon cancer patients [77], but also many other markers, while not always with completely reliable data (reviewed in [21]). Two recent studies on PSA are a milestone example on the importance of the reappraisal of exosomal expression of known tumor biomarkers. The first one showed the increased expression of PSA in exosomes that are purified from both prostate cancer cells under acidic culture conditions and plasma of prostate cancer patients [35]. More importantly, a prospective clinical study has shown that the plasmatic levels of exosome expressing PSA was significantly higher in patients with prostate cancer as compared to both patients with BPH and healthy individual patients [36]. These data were obtained by implementing two different techniques, both using monoclonal antibodies against exosome antigens and PSA, i.e., IC-ELISA and NFC. The prospective clinical study was performed in 240 individuals, including 80 PC patients, 80 BPH patients, and 80 healthy CTR [36]. The statistical analysis showed that pooling together the two analysis, the PSA exosome determination got to 96% of sensitivity and 100% specificity in distinguishing PC from BPH patients, while the IC-ELISA alone showed 98.57% of sensitivity and 80.28% of specificity in distinguishing PC patients from BPH patients, and 100% of both sensitivity and specificity in distinguishing PC patients from healthy individuals [36]. This last result has suggested that IC-ELISA may be exploited in future screening tests performed in young men to the purpose of getting to a reliable early diagnosis of prostate cancer with a non-invasive technique.

In addition to plasma, urine is another body fluid that can be easily exploited in the clinical management of cancer patients, particularly in patients with genitourinary tract malignancies. Interestingly, proteomic analysis of urinary EVs may represent a valuable diagnostic tool in certain kidney pathologies [90]. The authors showed that polycystin-1 and polycystin-2 expression, together with that of other Ca2+-binding proteins (annexin A1, annexin A2, protein S100-A9, protein S100-A8, and retinoic acid-induced protein 3) expressed in EVs preparations from patients’ urines are significantly altered in kidney diseases, including tumors [90].

Exosomes of the prostate epithelium that are released into semen or urine (prosteasome) also contain important molecular information related to certain types of tumors [91,92]. For example, analysis of urine-derived exosomes of prostate cancer patients identified tumor biomarkers, such as prostate-specific antigen and *PCA3* [86,92,93,94,95]. Increased level of d-catenin has shown in exosomes preparations obtained from both prostate cancer patients’ urine and PC-3 cell culture supernatants [96]. Some potential exosome-associated biomarkers related to the EGF receptor signaling mechanism were shown in bladder cancer as well [97,98].

An entirely new and exciting area is the identification of tumor-associated nucleic acids within the exosome preparation obtained from patients’ body fluids. The reports suggesting that RNAs are pretty detectable in exosome samples are increasing, also suggesting that within exosomes the RNAs are protected from degradation by ribonucleases enriching the human body fluids [99].

## 6. Circulating Exosomal miRNAs as Tumor Biomarkers

Besides the protein cargo, the exosomes isolated from different body fluids contain a significant amount of nucleic acids, such as mRNA, miRNA, long non-coding RNA (lncRNA), as well as DNA. Starting from Valadi’s discovery of exosomal mRNAs and miRNAs [15], exosomal miRNAs have been extensively studied as they are the most abundant in exosomes and EVs [100]. Important differences have been shown in exosomal miRNAs composition and amount between cancer patients and healthy individuals, supporting their use as potential non-invasive clinical biomarkers [101] (summarized in Table 2).

However, the use of exosomal miRNA in clinical oncology has been to date limited by technical and analytical biases that affect the yield, integrity, and purity of the exosomal miRNA [2,102]. Indeed, the miRNAs in biological fluids are either packaged in the vesicles or associated with RNA-binding protein (e.g., Argonaute 2) or with lipoprotein complexes (mostly HDL and LDL) [2,102,103]. During ultracentrifugation for exosomes isolation, circulating non-exosomal RNA are pelleted together with exosomal preparations with subsequent RNA contamination in EVs preparations [103]. One possible approach to solve this drawback could be to immunocapture exosome/EVs before carrying on with miRNA detection. In fact, through this technique, exosomes may be attached to plastic plates covered with antibodies recognizing specific exosomal markers (or even a specific tumor marker if available) and perform miRNAs characterization on the attached exosome/EVs population. It appears a feasible technical approach that will make a more reliable RNA characterization on EVs purification from patients’ samples.

**Table 2:** Detection of exosomal microRNAs as tumor biomarkers in serum and plasma.

Pathology	Biomarker	Source	Isolation Method	Potential Use	Reference
Prostate cancer	miR-141, miR-375	Serum	Filtration-based capture of exosomes	Diagnosis/Stage Determination	[104]
	miR-1290, miR-375	Plasma	Polymeric precipitation	Prognosis	[105]
	miR-141	Serum	Polymeric precipitation	Diagnosis	[106]
Colorectal cancer	let-7a, miR-1229, miR-1246, miR-150, miR-21, miR-223, miR-23a	Serum	UC	Early Diagnosis	[107]
	miR-19	Serum	UC, Polymeric precipitation	Prognosis	[108]
	miR-4772-3p	Serum	Polymeric precipitation	Prognosis for recurrent stage II, III	[109]
	miR-21	Plasma	UC	Prognosis	[110]
	miR-221	Plasma	UC	Prognosis	[111]
Ovarian cancer	miR-21, miR-214, miR-200a, miR-200b, miR-200c, miR-203, miR-205, miR-141	Serum	Modified magnetic activated cell sorting (MACS) procedure	Early Diagnosis/Prognosis	[112]
	miR-373, miR-200a, miR-200b, miR-200c	Serum	Polymeric precipitation	Diagnosis/Prognosis	[113]
	miR-21, miR-100, miR-200b, miR-320	Plasma	Polymeric precipitation	Diagnosis	[114]
Breast cancer	miR-101, miR-372, miR-373	Serum	Polymeric precipitation	Diagnosis	[115]
	miR-1246, miR-21	Plasma	UC, Polymeric precipitation	Diagnosis	[116]
Lung cancer	miR-151a-5p, miR-30a-3p, miR-200b-5p, miR-629, miR-100, miR-154-3p	Plasma	Polymeric precipitation	Early Diagnosis	[117]
Esophageal Squamous Cell Carcinoma	miR-21	Plasma	Polymeric precipitation	Early Diagnosis/Therapy	[118]
Laryngeal Squamous Cell Carcinoma	miR-21and HOTAIR (lncRNA)	Serum	Polymeric precipitation	Diagnosis/Prognosis	[119]
Pancreatic cancer	miR-17-5p, miR-21	Serum	UC	Diagnosis/Prognosis	[120]
	miR-1246, miR-4644, miR-3976, miR-4306	Serum	UC, Density gradient centrifugation	Diagnosis	[74]
	miR-10b, miR-21, miR-30c, miR-181a, miR-let7a	Plasma	UC	Diagnosis	[121]
	miR-191, miR-21, miR-451a	Serum	Polymeric precipitation	Diagnosis	[122]
	miR-451a	Plasma	UC	Prognosis	[123]
Gastric cancer	miR-423-5p	Serum	Polymeric precipitation	Diagnosis/Prognosis	[124]
Hepatocellular carcinoma	miR-18a, miR-221, miR-222, miR-224	Serum	Polymeric precipitation	Diagnosis	[125]
	miR-718	Serum	UC	Diagnosis/Prognosis/Recurrence	[126]

UC, ultracentrifugation.

## 7. The Strategic Role of Immunocapture-Based Approach in the Development of Exosome-Based Diagnostic Approach in Cancer

When our group described for the first time the immunocapture-based approach in exosome-based cancer diagnostic [16] (patent Exo-test, PCT/EE2009/000001), it received many criticisms in the ISEV community, mostly based on the use of anti-Rab5 antibodies to capture the EVs. However, in the last five years, many groups across the world have reported both pre-clinical and clinical data obtained with the same approach, and thus, supporting an agreed use of this methodology in cancer diagnostics [127,128]. We had the chance to compare a modified IC-ELISA to both NFC and NTA in their potential clinical relevance. IC-ELISA showed many advantages as compared to both the other techniques inasmuch it allows at the same time to quantify and characterize the clinical sample, and to include broader populations of EVs in terms of size, from nano to micro [35,36].

Moreover, differently to NTA, IC-ELISA evaluated the expression of a broad spectrum of biomarkers, including both those EVs specific and those that may represent either specific or surrogate tumor biomarkers [16,35,36,56] (Table 3), and most of all, in a considerable amount of clinical sample at the same time.

The IC-ELISA has the key advantage to be exportable in the majority of the clinical laboratories worldwide, needing to be implemented with the use of an ultracentrifuge that presently is the most accepted technical approach to obtain EVs and exosomes from either pre-clinical or clinical samples [2]. The immunocapture-base technique may also represent a valuable new tool for differentiating EVs through their protein expression, and thus, allowing them to perform nucleic acid analyses in different EVs subpopulation, and this could be of paramount importance for clinical research based on micro-RNAs studies [99].

## 8. Conclusions

Incorporating IC-ELISA technology into a platform for exosome-associated mRNA analysis is expected to enable detection and quantification of plasma RNAs of well-defined tumor origin, providing highly sensitive and specific assessment while avoiding some of the ‘noise’ that hampers quantitative real-time PCR and microarray analysis of whole-body fluids. Besides, exosomes can be enriched in mRNAs and miRNAs, which are hardly detectable in the parent tissue where their signal is covered by the presence of a higher number of molecules. Thus, exosomes can well represent the source for the identification of novel disease-associated markers.

Altogether these data suggest that quantification and characterization of EVs in human body fluid may be highly helpful as a new non-invasive diagnostic tool for the clinical management of cancer patients. The different expression of tumor biomarkers in circulating exosomes may lead to early diagnosis, to improve the tumor staging, as well as to evaluate the progression of the disease. In fact, the clinical study showing the relevance of exosomes expressing PSA in prostate cancer diagnosis suggests that future clinical investigation should also be aimed at verifying a potential new role of the old tumor biomarkers when expressed on exosomes. However, further clinical studies are needed to validate the use of either plasmatic or other body-fluids derived exosomes in the clinical practice, with considerable advantages both for patients, avoiding or limiting unnecessary invasive procedures, and hopefully significantly reducing the costs of the public health.

## Figures and Tables

**Table 1:** Protein tumor markers on plasmatic exosomes.

Pathology	Exosomal Proteins (Biomarkers)	Potential Use	Source	Isolation Method	References
Melanoma	Caveolin-1	Diagnosis	Plasma	UC	[16]
	HSP70, HSP90	Prognosis	Plasma	UC, Density gradient centrifugation	[57]
	MIA, S100B	Diagnosis/Prognosis	Serum	Polymeric precipitation	[58]
Prostate Cancer (Pca)	PSA	Screening/Early Diagnosis	Plasma	UC	[35,36]
	CA IX	Diagnosis	Plasma	UC	[51]
	Survivin	Early Diagnosis	Plasma	UC	[59]
	Exosomes levels	Diagnosis/Prognosis/Disease surveillance	Plasma	UC	[60]
	PTEN	Diagnosis	Plasma	UC	[61]
Ovarian Cancer	EpCAM, CD24, CA-125	Diagnosis	Plasma	Microfluidic chip	[62,63,64]
	TGF-beta1, MAGE3/6,	Diagnosis/Prognosis/Therapy monitoring	Plasma	UC	[65]
Breast cancer	Breast cancer resistance protein (BCRP)	Prognostic biomarker	Plasma	UC	[66]
	Her2	Diagnosis/Molecular classification	Plasma	Microfluidic chip	[67]
		Trastuzumab resistance/Tumor aggressiveness	Plasma	UC	[68]
	Glypican-1	Screening/Diagnosis	Serum	UC	[69]
	Fibronectin	Early Diagnosis	Plasma	ELISA	[70]
	Periostin	Diagnosis	Plasma	UC	[71]
	Del-1	Early Diagnosis/Prognosis	Plasma	UC	[72,73]
Pancreatic cancer	CD44v6, Tspan 8, EpCAM, CD104	Diagnosis/Prognosis	Serum	UC	[74]
	Glypican-1	Screening/Diagnosis	Serum	UC	[69]
Colorectal cancer	Hsp60	Diagnosis/Therapy	Plasma	UC	[55]
	TSAP6/CEA	Diagnosis/Prognosis	Plasma	UC	[75]
	Glypican-1	Diagnosis/Therapy target (treatment)	Plasma	Immunocapture assays	[76]
	CEA	Diagnosis	Serum	UC/Polymeric precipitation	[77,78]
	CD147	Diagnosis	Serum	UC	[79]
			Plasma	UC	[80]
Gastric cancer	GKN1	Diagnosis/Prognosis	Serum	Polymeric precipitation	[81]
	TGF-β1	Diagnosis	Plasma	UC	[82]
Lung cancer	NY-ESO-1	Prognosis	Plasma	Extracellular Vesicle Array	[83]
Oral Squamous Cell Carcinoma	CAV-1	Follow up	Plasma	UC	[56]
Hematological tumors	CD9, CD13, CD19, CD30, CD38, CD63	Diagnosis	Serum	UC	[84]

UC, ultracentrifugation.

**Table 3:** Detection of exosomal tumor biomarkers in body fluids by immunocapture-based ELISA (IC-ELISA).

Pathology	Biomarker	Approach Used	Source	Potential Use	Clinical Study Size (*N*)	Reference
Melanoma	Caveolin-1	IC-ELISA	Plasma	Early Diagnosis	Control *N* = 58; Disease *N* = 90	[16]
	MIA, S100B	IC-ELISA	Serum	Diagnosis/Prognosis	Control *N* = 25; Disease free *N* = 18; Disease (stage IV) *N* = 53	[58]
	MT-CO2, COX6c	IC-ELISA	Plasma	Diagnosis	Control *N* = 6; Disease *N* = 21	[127]
Prostate cancer	PSA	IC-ELISA, NSFC	Plasma	Screening/Early diagnosis	Control *N* = 15; BPH *N* = 15; Disease *N* = 15	[35]
					Control *N* = 80; BPH *N* = 80; Disease *N* = 80	[36]
	Survivin	IC-ELISA	Plasma	Early diagnosis	Control *N* = 8; BPH *N* = 20; Disease *N* = 39	[59]
	ephrinA2	IC-ELISA	Serum	Diagnosis	Control *N* = 20; BPH *N* = 21; Disease *N* = 50	[129]
Bladder cancer	TACSTD2	Flow cytometry, LC-MS/MS, LC-MRM/MS, IC-ELISA	Urine	Diagnosis	Control *N* = 81; Disease *N* = 140	[130]
Colorectal cancer	Hsp60	IHC, IC-ELISA, IEM	Plasma	Diagnosis/Therapy	Control *N* = 40; Disease *N* = 57	[55]
	CEA	IC-ELISA	Serum	Diagnosis	Control *N* = 8; Disease *N* = 116	[78]
	CPNE3	IC-ELISA	Plasma	Diagnosis/Prognosis	Control *N* = 32; Disease *N* = 92	[131]
Ovarian cancer	MT-CO2, COX6c	IC-ELISA	Plasma	Diagnosis	Control *N* = 6; Disease *N* = 62	[127]
Breast cancer	Fibronectin	IC-ELISA	Plasma	Early Diagnosis	Control *N* = 70; After surgery *N* = 40; Benign disease *N* = 55; Noncancerous diseases *N* = 80; Disease *N* = 240	[70]
	Del-1	IC-ELISA	Plasma	Diagnosis/Monitoring/Treatment	Control *N* = 81; After surgery *N* = 50; Benign disease *N* = 64; Noncancerous diseases *N* = 98; Disease *N* = 269	[72]
					Control *N* = 22; Disease *N* = 114	[73]
	MT-CO2, COX6c	IC-ELISA	Plasma	Diagnosis	Control *N* = 6; Disease *N* = 13	[127]
Lung squamous cell carcinoma	14-3-3ζ	IC-ELISA	Plasma	Diagnosis	Control *N* = 17; Disease *N* = 17	[132]
Oral Squamous Cell Carcinoma	CAV-1	IC-ELISA	Plasma	Follow up	Disease *N* = 10 (before and after surgery)	[56]

BPH, benign prostatic hyperplasia; IC-ELISA, immunocapture based-enzyme-linked immunosorbent assay; IEM, immunoelectron microscopy; IHC, immunohistochemistry; LC-MS/MS, liquid chromatography–tandem mass spectrometry; LC-MRM/MS, liquid chromatography–multiple reaction monitoring-mass spectrometry; NSFC, nanoscale flow-cytometry.
